# The Maturing Development of Gut Microbiota in Commercial Piglets during the Weaning Transition

**DOI:** 10.3389/fmicb.2017.01688

**Published:** 2017-09-04

**Authors:** Limei Chen, Yuesong Xu, Xiaoyu Chen, Chao Fang, Liping Zhao, Feng Chen

**Affiliations:** ^1^State Key Laboratory of Microbial Metabolism, School of Life Sciences and Biotechnology, Shanghai Jiao Tong University Shanghai, China; ^2^Key Laboratory of Systems Biomedicine (Ministry of Education), Shanghai Center for Systems Biomedicine, Shanghai Jiao Tong University Shanghai, China

**Keywords:** piglet, development, gut microbiota, weaning, *Fusobacterium*

## Abstract

Early weaned piglets are vulnerable to diarrhea because of weaning stress and immaturity of intestinal tract. Compelling evidence suggests that gut microbiota is vital to host health. However, it is not well understood on the composition and succession of piglet gut microbiota during the weaning transition. In our two trials, total 17 commercial piglets were studied in a pig farm in Jiangxi Province, China. Fresh feces were collected for four times (10 days before weaned, weaned day, 10 days after weaned, 21 days after weaned) by rectal massage. Fecal bacterial composition was assessed by 16S rRNA gene V3–V4 regions sequencing by Illumina Miseq platform. The results showed that the gut microbiota of piglets shifted quickly after weaned and reached relatively stable level in 10 days after weaned. The alpha diversity increased significantly with the age of piglets. The microbiota of suckling piglets was mainly represented by *Fusobacterium, Lactobacillus, Bacteroides, Escherichia/Shigella*, and *Megasphaera*. This pattern contrasted with that of *Clostridium sensu stricto, Roseburia, Paraprevotella, Clostridium* XIVa, and *Blautia*, which were major representative genera after weaned. In summary, we delineated the development of piglet gut microbiota during the weaning transition. This study helps us understand the maturing development of gut microbiota in commercial piglets.

## Introduction

The human gastrointestinal tract is the home to microbes which is approximate 10-times to our own cells, and thousands of bacterial phylotypes colonize the entire length of the gut with a collective genome that contains at least 100-times as many genes as our own genome (Gill et al., 2006; Qin et al., 2010). Accumulating evidences suggest that gut microbiota plays a vital role in host health. For example, recent studies have shown that gut microbiota was tightly associated with the incidence of many chronic diseases, such as obesity, diabetes, and colon cancer (Wang et al., 2012; Zhao, 2013; Xu et al., 2015; Zhang C. et al., 2015). Moreover, early-life establishment of infant gut microbiota sets the stage for adult microbiome and has prolonged influence on host health (Turnbaugh et al., 2009; Houghteling and Walker, 2015). Early weaned piglets are vulnerable to infect diseases because of weaning stress and immaturity of intestinal tract (Kim J.C. et al., 2012). Due to the similarities between pig and human gastrointestinal tract, studies with pigs as model also have drawn a great interest (Shen et al., 2010; Heinritz et al., 2013). Thus researches on the composition and succession of gut microbiota of piglets are necessary for both pig health and human health.

Although much studies about the development of gut microbiota in adult pigs and its relationship with antibiotics treatment have been explored (Kim et al., 2011; Kim H.B. et al., 2012; Looft et al., 2012), few studies have been focused on the development of gut microbiota during suckling and weaning period. Weaning is a special and important event for piglets, and is a challenge to piglet gut physiology (Lallès et al., 2007). A study about gradual changes of gut microbiota in weaned miniature piglets has been done recently (Hu et al., 2016), while it was small in scale and did not study the gut microbiota of piglets during suckling period. Thus, further research on the development of gut microbiota in piglets is necessary and of great significance.

The work presented here was designed to better understand the development of gut microbiota in piglets during the weaning transition. The gut microbiota was studied from 17 piglets across 4 age strata by 16S rRNA gene V3–V4 regions sequencing with Illumina Miseq platform. In this study, we characterized the composition and dynamic shifts of piglet gut microbiota, identified the phylogenetic core regardless of the wide gut microbiota structural variation across age, and explored the potential interaction among microbes in piglets.

## Materials and Methods

### Animals and Sample Collection

Two replicated trials (named T1 and T2, respectively) were conducted on a commercial pig farm located in Yichun city, Jiangxi province, China. Six litters of piglets were involved in this study. Two or three piglets from each litter were randomly selected and marked. A total of 17 two-week-old piglets (Duroc × [Landrace × Yorkshire]) with similar body weight were studied (T1, *n* = 8. male:female = 5:3. T2, *n* = 9 male:female = 4:5). All piglets stayed with their mothers during suckling period. Piglets in the same trial were transferred into the same pen after weaned. Corn and soybean meal as main sources of energy and amino acid were used in diet (Supplementary Table 1). Feed and water were offered *ad libitum*. None of piglets received antibiotic therapy during the period from birth to the end of the study. Fresh feces from all piglets were collected by rectal massage at 10 days before weaned (b10d), weaned day (00d), 10 days after weaned (10d), and 21 days after weaned (21d). All fecal samples were transported to lab stored on dry ice and stored at -80°C until use at lab. All operations of this animal experiment were approved by the Institutional Animal Care and Use Committee of Jiangxi ZhengYu (NO: 2016-007) and carried out in accordance with its guidelines.

### DNA Extraction

DNA extraction from fecal samples was conducted as described previously (Godon et al., 1997). Briefly, bacterial cells were broken with *N*-Lauroylsarcosine, and followed by shocking with 0.1 mm class beads (No.11079101, BioSpec Products, United States) by TissueLyser II (Schwingmuhle TissueLyser 2, Germany). Isopropanol was used to precipitate nucleic acid after removing impurities and inhibitors. The nucleic acid were then treated with RNase to degrade RNA and ethyl alcohol was used to purify and precipitate DNA. The quality and concentration of DNA were detected by agarose gel electrophoresis and BioDrop (Spectrophotometer, Cambridge, England).

### V3–V4 Regions in 16S rRNA Gene Sequencing

A sequencing library of the 16S rRNA gene V3–V4 regions was established according to the protocol provided by Illumina^1^ with some modifications. Phanta Max Super-Fidelity DNA polymerase (P505-d1, Vazyme, Nanjing, China) was used for two steps of amplification. For the Amplicon PCR (amplification of 16s rRNA gene V3–V4 region), the 25-μl reaction mix contained 2x Phanta Max Buffer, 10 mM dNTP Mix, 1 μM of each specific primer for V3–V4 region of 16S rRNA gene as described in protocol, 0.5 U of Phanta Max Super-Fidelity DNA polymerase and 10 ng microbial DNA. PCR condition were pre-denaturation at 95°C for 3 min: 21 cycles of 95°C for 30 s, 55°C for 30 s, 72°C for 30 s: and a final 5 min extension at 72°C. The Amplicon PCR products were detected by 1.2% agarose gel electrophoresis and purified by Agencourt AMPure XP (A63881, Beckman Coulter, United States). For the Index PCR (attachment of dual indices and Illumina sequencing adapter using the Nextera XT Index Kit), the 25-μl reaction mix contained 2x Phanta Max Buffer, 10 mM dNTP Mix, 2.5 μl of each N7 and S5 Index primers as described in protocol, 0.5 U of Phanta Max Super-Fidelity DNA polymerase, and 2.5 μl purified product of the Amplicon PCR. The program for Index PCR was the same as Amplicon PCR except that the cycle number was reduced to 8. The purification of Index PCR products was also conducted with AMPure XP beads according to the protocol. The purified products were mixed at equal ratio for sequencing using the Illumina Miseq System (Illumina Inc., United States).

### Analysis of Sequencing Data

The first base of both forward and reverse ends of the same read were truncated because *Q*-value was no more than 2. The pair of reads were merged into a complete read if they had a minimum overlap of 50 bp. These kind of reads were discarded unless longer than 399 bp with an expected error of no more than 0.5 (Edgar, 2010). High-quality reads were dereplicated into unique sequences and sorted by decreasing abundance, and singletons were discarded. Default of Uparse (Edgar, 2013) were used to pick representative non-chimeric operational taxonomic units (OUT) sequences. Further reference-based chimera were detected by UCHIME (Edgar et al., 2011) based on the RDP classifier training database (v11) (Cole et al., 2014). The OTU table was achieved by mapping high-quality reads to the remaining OTUs with the Usearch (Edgar, 2010) global alignment algorithm at a 97% cutoff. The sequences of all the samples were downsized to 13000 (1000 permutation) to avoid bias caused by the difference in sequencing depth. All subsequent analysis was conducted against the QIIME platform (version 1.8) (Caporaso et al., 2010). The observed OTUs and Shannon index were used to assess the alpha diversity of each sample. Phylogenetic tree was built by FastTree with representative sequences of each OTU and these representative sequences were subjected to the RDP classifier to determine the phylogeny with a bootstrap cutoff of 80% (RDP database version 2.11).

Since T1 and T2 showed similar gut microbiota structure and change trend, Random forest (Breiman, 2001) and co-abundance network were performed based on pooled data from two trials. Specific bacterial phylotypes which contributed to the segregation of gut microbiota were identified by Random forest models (Breiman, 2001). Group pairs which has a significant difference [*P* < 0.05, Permutational Multivariate Analysis of Variance (PERMANOVA) based on Bray–Curtis distance and weighted UniFrac distance] were included for random forest discrimination. Models with ratio of baseline error to estimated error more than 2 were considered successful. The mean decrease in accuracy of discrimination determined the importance of an OTU, and OTUs with a value greater than 0.002 were considered as key OTUs.

The SparCC algorithm (Friedman and Alm, 2012) was used to calculate the correlation among 85 key OTUs with a bootstrap procedure repeated 100 times and then the correlation was visualized into a network diagram. Eighty-five key OTUs clustered into 10 co-abundance group (CAGs) with the Ward clustering algorithm and PREMANOVA (9999 permutations, *p* < 0.01) based on SparCC correlation coefficient by R program (v2.11.1).

### Statistical Analysis of Microbiota Data

The Mann–Whitney test and Wilcoxon signed-rank test were used to analyze the variance of microbiota data. Interindividual and intraindividual structural variations were tested by student’s *t*-test with 1,000 Monte Carlo permutations based on Bray–Curtis and weighted UniFrac distances. To determine whether the gut microbiota structure was significant different between different age phases, PERMANOVA was performed based on the Bray–Curtis and weighted UniFrac distances in the R “vegan” package (Oksanen et al., 2013) with 9,999 permutations. The multiple comparisons were adjusted as described by Benjamini and Hochberg (1995). Differences were considered significant when *P*-value (corrected) < 0.05. All statistical analyses were carried out by GraphPad Prism (v 5.01), R (v2.11.1) software and Matlab R2014b.

### Accession Number

The 16S rRNA gene sequence information in this study was deposited in NCBI Sequence Read Archive (SRA) under accession number SRP102931.

## Result

### Overall Gut Microbiota Structure of Piglet

We collected a total of 68 piglet fecal samples during May–June in 2016. Illumina Miseq sequencing of the V3–V4 regions of bacterial 16S rRNA genes generated 2,377,625 high-quality sequences (594,957 unique sequences). After removal of chimeras, filtered high-quality sequences were grouped into 1348 OTUs.

**FIGURE 1:**
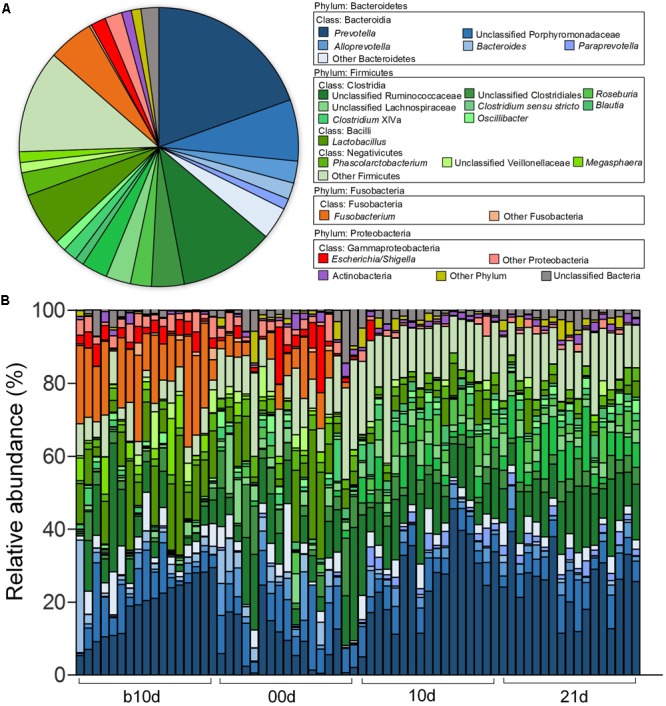
Genus-level gut microbiota composition of the piglets. **(A)** The dominant genera (1% of the total sequences) in the 68 fecal samples of piglets. **(B)** Relative contribution of these dominant genera in each sample. Samples are arranged according to the proportion of *Prevotella* (the most dominant genus) in b10d and paired sample in other three sample collected time points.

Across all the samples, 97.93 and 46.94% of the total sequences were assigned into 21 phyla and 151 genera, respectively. Unclassified bacteria occupied about 2.07% of the total sequences. Firmicutes and Bacteroidetes constituted the two predominant phyla in the piglet gut microbiota (contributing 50.36 and 36.08% of the total sequences, respectively) (**Figure 1A**). Besides Firmicutes and Bacteroidetes, Fusobacteria (5.52%), Proteobacteria (3.74%), and Actinobacteria (1.12%), which occupied more than 1% of the total sequences were considered as predominant phyla as well. At the genus level, there were 14 taxa that their abundance were more than 1% of the total sequences. *Prevotella* was the most predominant genus (19.50% of the total sequences) in piglet gut microbiota (**Figure 1A**). *Alloprevotella* (2.55%), *Bacteroides* (1.91%), and *Paraprevotella* (1.24%) were other three dominant genera in Bacteroidetes. In the class Clostridia from Firmicutes, unclassified Ruminococcaceae was most abundant, occupied 11.00% of the total sequences (**Figure 1A**). Other detected dominant genera which belonged to class Clostridia included *Clostridium sensu stricto* (3.00%), *Roseburia* (2.43%), *Clostridium* XlVa (1.64%), *Oscillibacter* (1.26%) and *Blautia* (1.04%). *Lactobacillus* (6.28%) was the only dominant genus in Bacilli (**Figure 1A**). *Phascolarctobacterium* and *Megasphaera*, two genera from Negativicutes, composed 2.69 and 1.25%, respectively (**Figure 1A**). *Fusobacterium*, which belonged to Fusobacteria, occupied 5.27% of the total sequences (**Figure 1A**). In Proteobacteria, *Escherichia/Shigella* was the only dominant genus which occupied 1.74% (**Figure 1A**). When explored genus-level gut microbiota composition in T1 and T2 independently, it showed similar pattern between two trials (Supplementary Figures 1A,B). Genus-level gut microbiota composition in each sample indicated that piglets with different age showed wide variation in bacterial community composition except piglets between 10d and 21d (**Figure 1B**).

### Maturing-Related Overall Structural Changes of Gut Microbiota

**FIGURE 2:**
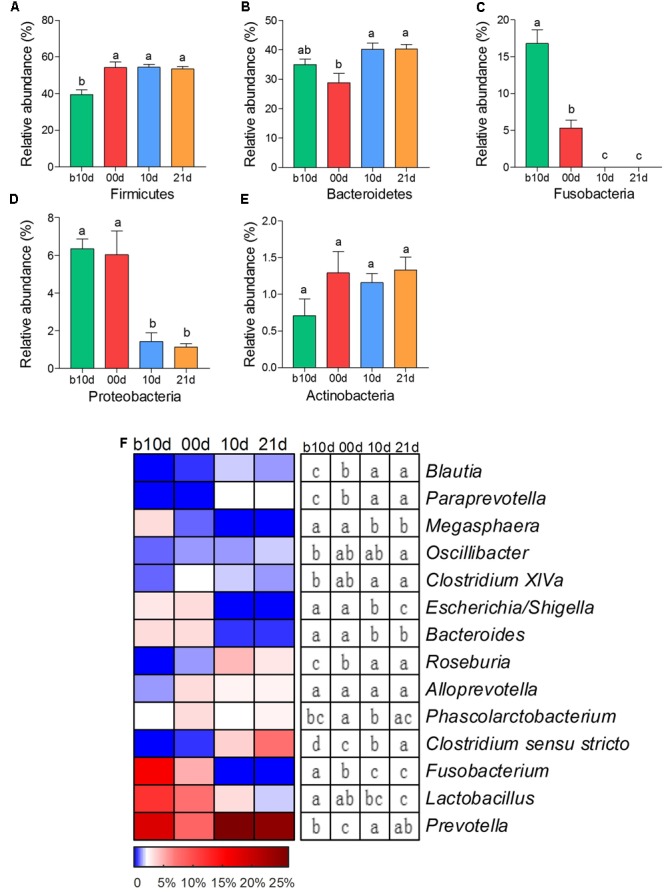
The shifts of 5 predominant phyla and 14 predominant genera in the gut bacterial compositions of piglets during the weaning transition. **(A)** Firmicutes **(B)** Bacteroidetes **(C)** Fusobacteria **(D)** Proteobacteria **(E)** Actinobacteria. **(F)** The change in the relative abundance of predominant genera as piglets aged. The color of the spots in the panel represents the relative abundance of the predominant genera. Mean values ± SEM are shown. Different letters above the bar or in boxes denote significant difference between groups tested by paired sample Wilcoxon signed-rank test and adjusted by FDR.

As the two most abundant phyla, Firmicutes and Bacteroidetes showed significant increases in the relative abundance as the piglets aged (**Figures 2A,B**). However, the relative abundances of other two phyla, Fusobacteria and Proteobacteria, showed remarkable decreases with age of piglets (**Figures 2C,D**). Actinobacteria was the only predominant phylum that revealed no noteworthy shift in the relative abundance (**Figure 2E**). At the genus level, 7 predominant genera showed significant increases as the piglets aged, including *Blautia, Paraprevotella, Oscillibacter, Clostridium* XIVa, *Roseburia, Clostridium sensu stricto*, and *Prevotella* (**Figure 2F**). Among these genera, *Prevotella* composed of about 25% of total sequences of each sample in weaned piglets on average. The relative abundances of *Megasphaera, Escherichia/Shigella, Bacteroides, Fusobacterium*, and *Lactobacillus* showed prominent decreases as piglets aged (**Figure 2F**). Notably, *Fusobacterium*, which was abundant in the gut of suckling piglets (the mean relative abundances were 16.10 and 4.97% in b10d and 00d, respectively), was almost absent after weaned. The mean relative abundance of *Lactobacillus* declined from 12.25 to 1.76% during investigated period.

**FIGURE 3:**
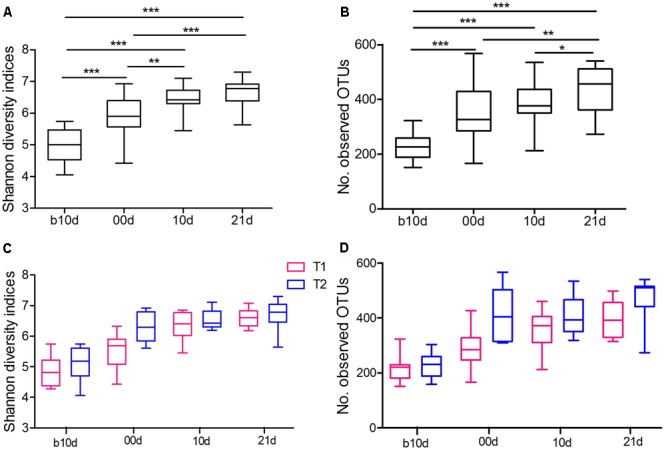
Variations in alpha diversity of the piglets. **(A)** Comparisons of Shannon diversity indices among different age piglets by paired sample Wilcoxon signed-rank test. **(B)** Comparisons of the number of observed OTUs among different age piglets by paired sample Wilcoxon signed-rank test. **(C)** Comparisons of Shannon diversity indices between T1 and T2 by Mann–Whitney test. **(D)** Comparisons of the number of observed OTUs between T1 and T2 by Mann–Whitney test. Both alpha diversity metrics were calculated upon the rarified OTU subsets, using 13,000 sequences per sample with 1,000 replications. In all panels, boxes represent the interquartile range (IQR) between the first and third quartiles. The lines inside boxes represent the median. Whiskers denote the lowest and highest values within 1.5 IQR from the first and third quartiles, respectively. ^∗^*P* < 0.05, ^∗∗^*P* < 0.01, ^∗∗∗^*P* < 0.001 (with FDR adjust).

To further detect the dynamics of gut microbiota structure during investigated period, we evaluated the alpha diversity of piglet gut microbiota. The piglets showed continuously increased alpha diversity with age according to Shannon index and the observed OTUs (**Figures 3A,B**). Shannon index, which reflects the species richness and evenness, increased markedly from b10d to 10d and showed no significant difference between 10d and 21d (**Figure 3A**). The observed OTUs, which reflects the richness of species, increased significantly from b10d to 00d and 10d to 21d (**Figure 3B**). There was no remarkable difference in Shannon index and the observed OTUs between the same age piglets of two trials (**Figures 3C,D**). When evaluated Shannon index and the observed OTUs independently in T1 and T2, the result confirmed the continuous increase of alpha diversity in piglet gut microbiota during investigated period (Supplementary Figure 2).

**FIGURE 4:**
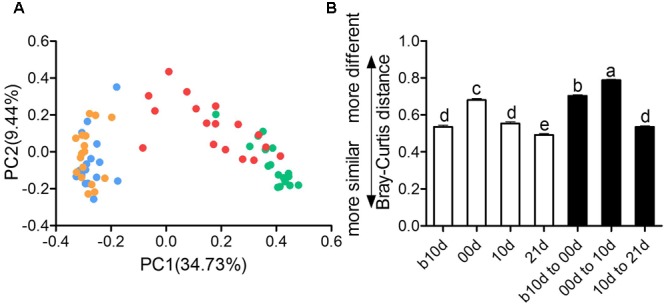
Inter- and intraindividual variations of the gut microbiota of the piglets. **(A)** Trajectory of the gut microbiota structure of each piglet across age based on Bray–Curtis distance. **(B)** Interindividual variations were determined by average Bray–Curtis distances between individuals in 10 days before weaned (b10d), weaned day (00d), 10 days after weaned (10d), or 21 days after weaned (21d), respectively, while intraindividual variations were determined by distances between paired b10d and 00d, 00d and 10d, and 10d and 21d samples, respectively. Mean values ± SEM are shown. Different letters above the bar denote significant difference tested by Student’s *t*-test with 1,000 Monte Carlo permutations.

The remarkable changes in composition and alpha diversity of the piglet gut microbiota across individuals and different ages led us to assess the extent of interindividual structural variations with each age and intraindividual variations across age. The principal coordinate analysis (PCoA) based on Bray–Curtis distance revealed that the gut microbiota of piglets showed obvious segregation from b10d to 10d but mingled together in 10d and 21d (**Figure 4A**). Moreover, the principal components analysis (PCA) and PCoA based on other distances revealed a similar shifting pattern (Supplementary Figure 3). PERMANOVA based on Bray–Curtis and weighted UniFrac distance demonstrated that gut microbiota structure of piglets changed significantly from b10d to 10d but showed no remarkable difference between 10d and 21d (Supplementary Table 2). While the alpha diversity was continue to increase, the interindividual Bray–Curtis distances between different individuals increased significantly during suckling period and decreased markedly during weaning period (**Figure 4B**). The interindividual variation in 00d was higher than that in b10d, 10d, and 21d. Furthermore, the gut microbiota of piglets within highest alpha diversity in 21d showed lowest interindividual structural variation. The significant changes in the gut microbiota structure from b10d to 10d were also verified by dramatic intraindividual variations from b10d to 00d and 00d to 10d, which were significantly higher than that from 10d to 21d (**Figure 4B**). Intraindividual variation from 10d to 21d was lower than interindividual variations in b10d, 00d, and 10d, in which also manifested that the gut microbiota structures of 10d and 21d were similar. Structural variations of interindividual and intraindividual based on Bray–Curtis distance evaluated in T1 and T2 independently, demonstrated the parallel result (Supplementary Figures 4A,B). Such interindividual and intraindividual structural variations were also supported by weighted UniFrac distance (Supplementary Figure 4C). The results of PCoA, PREMANOVA and inter- and intraindividual structural variations, suggest that the gut microbiota of piglets might reach relative stable and mature level in 10d.

**FIGURE 5:**
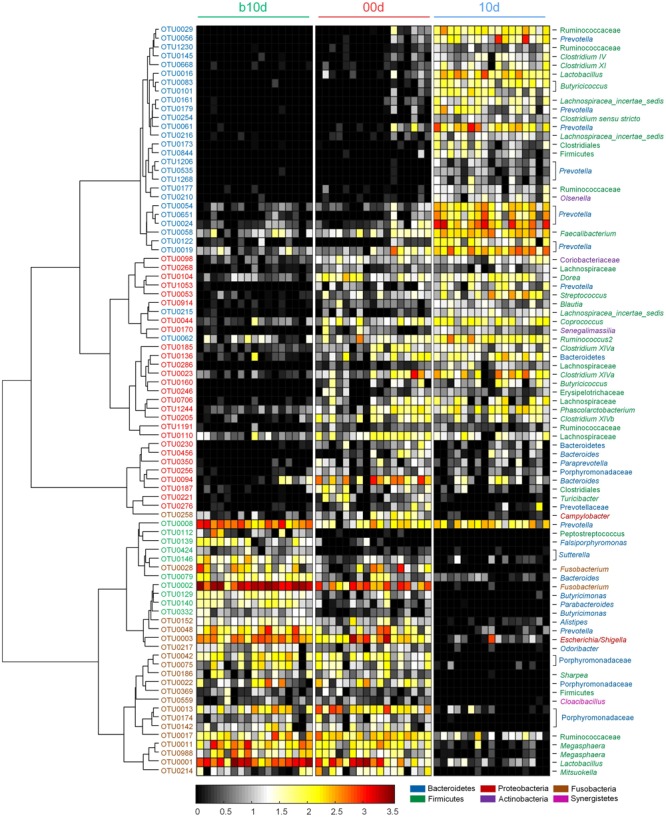
Heat map of 85 key OTUs responding to different age identified by random forest models. The color of the spots in the panel represents the relative abundance (normalized and log-transformed) of the OTU in each sample. The OTUs are organized according to their Spearman correlation coefficient. The OTUs increased in relative abundance from b10d to 00d are marked with red on the OTU id, whereas decreased are marked with green, and the OTUs increased in relative abundance from 00d to 10d are marked with blue on the OTU id, whereas decreased are marked with brown.

Random forest models identified 85 non-redundant OTUs as key OTUs, include 37 OTUs responding to age during suckling period and 49 OTUs responding to weaning (feature accuracy > 0.002, **Figure 5**, Supplementary Figure 5 and Table 3). During suckling period, 27 OTUs became more abundant in 00d than b10d and most of them belonged to Lachnospiraceae (**Figure 5**). In the contrary, the abundance of other 10 OTUs from Sutterellaceae, Fusobacteriaceae, and Porphyromonadaceae decreased from b10d to 00d. During weaning, 28 OTUs were enriched in 10d and these OTUs belonged to Ruminococcaceae and Prevotellaceae (**Figure 5**). However, 21 OTUs was less abundant or even absent in 10d than 00d. Most of these OTUs from Porphyromonadaceae, Erysipelotrichaceae, and Veillonellaceae. OTU2, which belonged to *Fusobacterium*, was the most abundant OTU in b10d (occupied 14.65% in each sample on average) but was almost absent in 10d, and it was the only OTU that contributed significantly to the establishment of both models (**Figure 5**). Detail taxonomy and dynamics information of these key OTUs were showed in Supplementary Table 4.

### The Genus and OTU Level Phylogenetic Core

**FIGURE 6:**
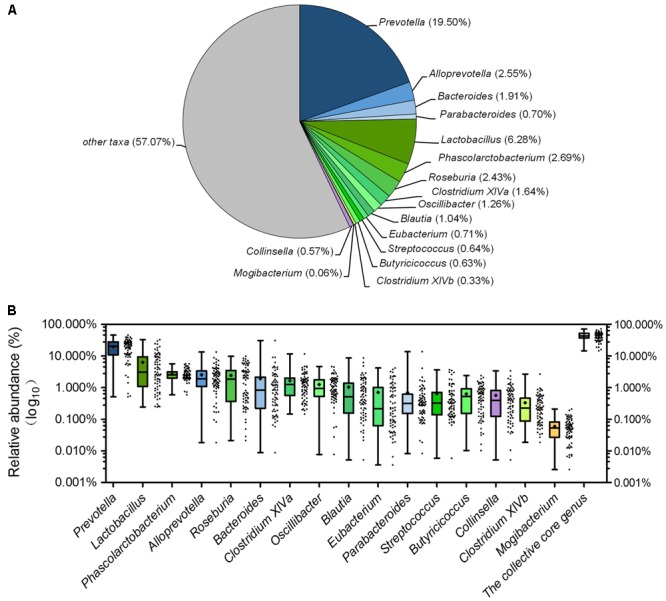
The piglets share a core gut microbiota composed of 16 bacterial genera. **(A)** The proportion of each genus in the total sequences. **(B)** The abundance distribution of the 16 genera and the collective core. Boxes represent the interquartile range (IQR) between the first and third quartiles. The lines and spots inside boxes represent the median and mean, respectively. Whiskers denote the lowest and highest values within 1.5 × IQR from the first and third quartiles, respectively.

Although there were significant differences in gut microbiota structure across age, core bacterial community which prevalent in samples may exert important function in gastrointestinal health. To identify this core bacterial community, any genus- or OTU-level phylotypes that occurred in all samples were picked out. Sixteen genera consisted of a genus-level phylogenetic core and occupied 42.93% of the total sequences (**Figure 6A**). Among the genera in this core, 4 genera belonged to Bacteroidetes, 11 from Firmicutes, and 1 (*Collinsella*) from Actinobacteria. Nine predominant genera belonged to this genus-level phylogenetic core, including *Prevotella, Alloprevotella, Bacteroides, Lactobacillus, Phascolarctobacterium, Roseburia, Clostridium* XIVa, *Oscillibacter*, and *Blautia* (**Figure 6A**). The collective core occupied >40% of the whole gut microbial community in more than 61.76% of samples but showed dramatic variations in abundance across all samples, which ranged from 14.62 to 71.54% of the whole gut bacterial community, regardless of age (**Figure 6B**).

At the OTU level, 12 OTUs were detectable in all samples, constituted an OTU-level phylogenetic core, composed of 15.79% of the total sequences (Supplementary Table 5). They represented the most prevalent OTUs, taken up only a minor part of all the 1348 OTUs (0.89%). The proportion of the collective core of OTUs in the whole gut microbial community ranged from 2.58 to 30.79% (Supplementary Figure 6). Among these OTUs, 5 OTUs occupied more than 1% of the total sequences in abundance, include 3 OTUs from Bacteroidetes (OTU7 and OTU21 which belonged to *Prevotella* occupied 3.55 and 2.35%, respectively, OTU10 which belonged to unclassified Porphyromonadaceae occupied 2.57%.) and 2 OTUs from Firmicutes (OTU6 which belonged to *Phascolarctobacterium* occupied 2.23% and OTU40 which belonged to unclassified Ruminococcaceae occupied 1.16%.) (Supplementary Table 5). OTU15, occupied about 0.88% of the total sequences, was an unclassified bacteria. Other 6 OTUs belonged to unclassified Bacteroidetes, unclassified Ruminococcaceae, unclassified Clostridiales, *Butyricicoccus, Blautia*, and *Collinsella*.

### Bacterial Interaction Network Analysis

**FIGURE 7:**
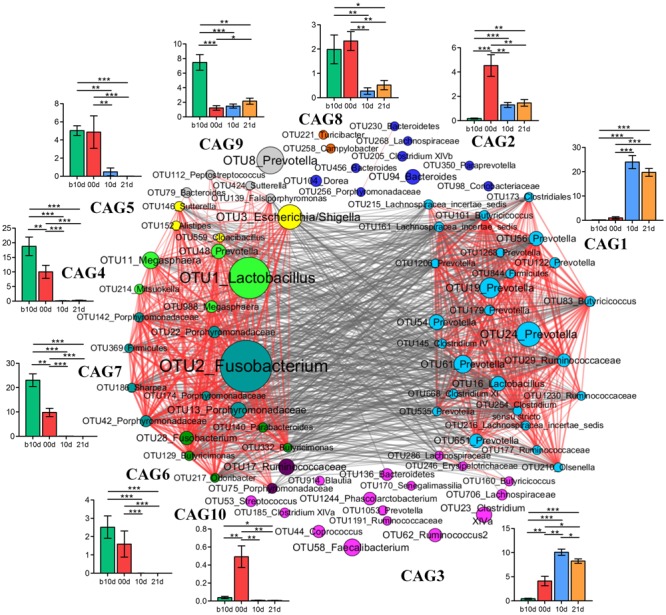
Co-variation in the gut microbiota of piglets during the weaning transition. OTU-level network diagram of 82 key OTUs responding to age. Node size indicates the mean abundance of each OTU. Lines between nodes represent correlations between the nodes they connect, the color saturation and line width indicating correlation magnitude: red represents positive correlation, gray represents negative correlation. Only lines corresponding to correlations with a magnitude greater than 0.5 are drawn. The OTUs are grouped into 10 CAGs by permutational multivariate analysis of variance (PERMANOVA) when *P* < 0.01. The plots show the abundance of each CAG on b10d, 00d, 10d, and 21d. Data in plots represent the total abundance of all OTUs in each CAG from each sample, which were then visualized by mean ± SEM. Paired sample Wilcoxon signed-rank test was used to analyze variations between two different time points. ^∗^*P* < 0.05, ^∗∗^*P* < 0.01, ^∗∗∗^*P* < 0.001 (with FDR adjust).

To identify the potential interaction that responded to age and weaning, the 85 key OTUs were clustered into 10 CAGs based on SparCC correlation coefficients (**Figure 7**). CAG4, 5, 6, and 7 were positively correlated with each other to form one cluster. Twenty-six OTUs clustered into CAG1 was the other main cluster. These two main clusters were negatively correlated with each other.

Weaning treatment decreased CAG2, 4, 5, 6, 7, 8, and 10 and enriched CAG1 and CAG3 (**Figure 7**). CAG4, 6, 7, and 10 were almost eradicated by weaning treatment. Notably, CAG4 and CAG7, which were abundant before weaned, decreased significantly as piglets aged from b10d to 10d. There were 5 OTUs in CAG 4, included *Lactobacillus* (OTU1), *Megasphaera* (OTU11, OTU988), *Mitsuokella* (OUT 214) and *Prevotella* (OTU48). A total of 8 OTUs from *Fusobacterium* (OTU2), Porphyromonadaceae (OUT142, OTU22, OTU186, OTU174, OTU42, OTU13), *Sharpea* (OTU186), and Firmicutes (OTU369) were contained in CAG7. OTU1 and OTU2 were the two most abundant OTUs and showed positive correlation with each other (*R* = 0.824).

## Discussion

Our study investigated the development of gut microbiota in piglets during the weaning transition. The result showed the composition and dynamic shifts of gut microbiota, phylogenetic core at genus and OTU level and potential interaction among key OTUs in piglets.

Consistent with previous studies, Firmicutes and Bacteroidetes were the two most dominant phyla in piglet gut microbiota, regardless of age (Kim et al., 2011; Looft et al., 2012; Hu et al., 2016). In our study, the most abundant phylum was Firmicutes during the whole investigated period. However, Bacteroidetes was the most abundant phylum in weaned piglet gut according to other two studies (Pajarillo et al., 2014; Hu et al., 2016). Accumulating evidences suggest that environment (Thompson et al., 2008) and diet (Pluske, 2013) affect gut microbiota structure of host. Thus these discrepancies in pig gut microbiota may attribute to the different environments and diets of experiments. Fusobacteria as the third abundant phylum during suckling, decreased significantly after weaned. This result was in line with previous studies (Pajarillo et al., 2014; Niu et al., 2015; Hu et al., 2016). Proteobacteria, which include a wide variety of pathogens, such as *Escherichia, Salmonella, Vibrio, Helicobacter*, showed significant decline after weaned. This observation was also observed in other four studies (Pajarillo et al., 2014; Niu et al., 2015; Zhao et al., 2015; Hu et al., 2016), indicating that the abundance of opportunistic pathogens might decrease as piglet gut microbiota maturing.

The abundances of *Fusobacterium* and *Escherichia/Shigella* sharply decreased from suckling period to weaning period. This observation was also found in the feces of infant (Kostic et al., 2015) and in line with other studies (Mann et al., 2014; Pajarillo et al., 2014; Mach et al., 2015). Interestingly, *Fusobacterium* was the second predominant genus in b10d which was indeed more abundant than *Lactobacillus*. Growing evidences suggest that *Fusobacterium* was closely correlated with cancer in humans and other diseases in animals (De Witte et al., 2017; Yang et al., 2017). This result suggesting that pathobiont species are commonly present in infant or inchoate piglet gastrointestinal tract, awaiting for potential opportunities to become pathogen. Because inchoate piglets are highly susceptible to various diseases, whether the proliferation of these potential pathogens has negative impacts for piglet health warrant further research. *Lactobacillus*, which was the third abundant genus in b10d, was predominant in piglet gut microbiota during suckling period. This observation was consistent with Mach’s study (Mach et al., 2015). Gut community of giant panda cubs were also dominated by *Lactobacillus* according to another recent study (Xue et al., 2015). However, *Bifidobacterium* was predominant in the human infant gut microbiota throughout the 1st year of life (Yatsunenko et al., 2012; Backhed et al., 2015). This was a distinct difference between pig or giant panda and human infant in gut microbiota. It remains to be determined the role of these microbes in the development of gut microbial community of the host. Among 7 predominant genera which significantly increased as piglets aged, three genera (*Blautia, Roseburia*, and *Prevotella*) revealed similar change tendency in miniature piglets during the early period after weaned (Hu et al., 2016) and infants from newborn to 12 months (Backhed et al., 2015). These microbes are efficient in degrading dietary fibers and producing short chain fatty acids (SCFA), indicating a shift toward more adult pig like intestinal environment associated with increased functional ability for carbohydrates degradation.

In our study, alpha diversity in gut microbiota of piglets showed continuous increase during investigated period. This result was congruent with previous studies (Pajarillo et al., 2014; Mach et al., 2015; Niu et al., 2015). However, other recent study showed continuously decreased alpha diversity until 11 days after weaned (Hu et al., 2016). In theory, a high level diversity provide “functional redundancy” that helps an ecosystem maintain its resilience, resistance and stability after environmental stresses (Naeem and Li, 1997; Konopka, 2009). A high diversity of gut microbiota is generally considered beneficial for host health (Turnbaugh et al., 2009; Le Chatelier et al., 2013) and it is also regard as the sign of mature gut microbiota. Weaning stress is a challenge for piglets. It seems that alpha diversity of gut bacterial community decreases first and then increases after weaned, and it shows augment from weaning to adulthood in pigs on the whole. Hu’s study weaned 3 days earlier than the present study, indicating that stronger weaning stress might slower the maturation of the gut microbiota in piglets and cause continuous decreased alpha diversity. Alleviating weaning stress as possible is a strategy for pig farm to improve the health condition of weaning piglets.

The present study illustrated that the gut microbiota structure of piglets changed significantly from 00d to 10d, whereas showed no remarkable difference between 10d and 21d. This change pattern was in accordance with a previous study, in which they divided the temporal change of gut architecture and function after weaned into two periods: an acute period (about 5 days) happening immediately after weaned and more progressive adaptative and maturational phase (Montagne et al., 2007). In our study, the interindividual Bray–Curtis distances between different individuals increased significantly during suckling period and decreased markedly during weaning period. This result suggested that gut microbiota structure of piglets became increasingly dissimilar during suckling period but was more and more similar after weaned. Newborn piglet gut microbiota has a simple and distinct microbial composition. Furthermore, immature gut microbiota is sensitive to environment factors and vulnerable to be disturbed. The speed of maturation of gut microbiota was also varied among each individual. Thus gut microbiota structure of piglets became dissimilar increasingly during suckling period. However, generally, the succession of piglet gut microbiota continues till the establishment of climax community (Isaacson and Kim, 2012), which contains microbes that remain in stable association with the host and have a relative population composition that is stable. Piglet gut microbiota in 10d may reach a climax community. Besides, more similar environment and the same dietary after weaned may also facilitate similar gut microbiota structure of piglets. Thus the microbial community structure became similar increasingly after weaned.

Despite of the wide gut microbiota structural variations across age, we characterized a phylogenetic core of gut microbiota which was made up of a small number of genus-level phylotypes. Most of these phylotypes were also prevalent in both western and eastern population (Zupancic et al., 2012; Zhang J. et al., 2015). Interestingly, except for *Mogibacterium*, which produced phenylacetate as a sole metabolic end product (Nakazawa et al., 2002) and occupied only 0.06% of the total sequences, other genera all can produce SCFA. Moreover, nine core predominant genera contain known SCFA-producing lineages. For example, *Prevotella* is a predominant SCFA-producing genus (Shah and Collins, 1990), *Lactobacillus* is a lactate producer, *Phascolarctobacterium* produces propionate via succinate fermentation (Watanabe et al., 2012), *Alloprevotella* mainly produces succinate and acetate (Downes et al., 2013), *Roseburia* is a butyrate producer (Duncan et al., 2002). SCFAs are major anions in the gut and absorbed rapidly by colonic epithelial cells. Compelling evidence suggests that these small molecules can protect the host against colonic diseases (Fukuda et al., 2011), improve the gut barrier function (Peng et al., 2009) and exhibit anti-inflammatory effects (Maslowski et al., 2009). The prevalence of this SCFAs producers across different age may indicate their essentiality for piglet health.

Co-abundance network among key OTUs showed that CAG4 and CAG7 were positive correlated with each other. OTU1 (*Lactobacillus*) and OTU2 (*Fusobacterium*) were the two most abundant OTUs which belonged to CAG4 and CAG7, respectively. Growing evidences suggest that *Fusobacterium* is involved in a wide spectrum of human diseases, such as oral infections, adverse pregnancy outcomes and gastrointestinal disorders (Han, 2015), and *Lactobacillus* is generally considered beneficial for host health. These two OTUs were positive correlated with each other which require to be further verified by well-designed experiment which focus on bacterial interaction. Network showed that different OTUs of the same genera such as 14 *Prevotella* were clustered into different CAGs, suggesting that different OTUs of the same genera might occupy different metabolic niches in the piglet gut ecosystem. OTUs of the same genera clustered into the same CAG, indicating that these OTUs might perform different functions in the gut ecosystem.

In the present study, we delineate the development of gut microbiota of piglets during the weaning transition. The gut microbiota shifted quickly after weaned and reached relative stable level in 10d in this study. This study may facilitate the researches of disease prevention during weaning. The results of this study also suggested the similarities and differences of gut microbiota between piglets and human beings. This may contribute to the researches of human gut microbiota with piglet animal model. In conclusion, this study help us understand the maturing development of gut microbiota in commercial piglets during the weaning transition.

## Ethics Statement

This study was carried out in accordance with the recommendations of Guidelines of Animal Care and Use Committee of Zhengyu, Animal Care and Use Committee of Jiangxi Zhengyu. The protocol was approved by the Animal Care and Use Committee of Jiangxi Zhengyu.

## Author Contributions

LZ and FC designed the study. LC and YX collected the piglet fecal sample. LC, XC, and CF performed the experiments. LC and FC analyzed the data. LC and FC wrote and revised the manuscript.

## Conflict of Interest Statement

The authors declare that the research was conducted in the absence of any commercial or financial relationships that could be construed as a potential conflict of interest.

## Footnotes

1 https://support.illumina.com/content/dam/illumina-support/documents/documentation/chemistry_documentation/16s/16s-metagenomic-library-prep-guide-15044223-b.pdf
