# Supplementary material for: The Maturing Development of Gut Microbiota in Commercial Piglets during the Weaning Transition
Source: Front Microbiol. 2017 Sep 4;8:1688. doi: 10.3389/fmicb.2017.01688 (PMC5591375; doi:10.3389/fmicb.2017.01688)
Supplement: Supplementary file 1 [file Presentation_1.pdf]

Supplementary Material

The Maturing Development of Gut Microbiota in Commercial Piglets during the Weaning Transition

Limei Chen, Yuesong Xu, Xiaoyu Chen, Chao Fang, Liping Zhao, Feng Chen*

*** Correspondence:** Feng Chen: cf2001@sjtu.edu.cn

1 Supplementary Figures and Tables

1.1 Supplementary Figures

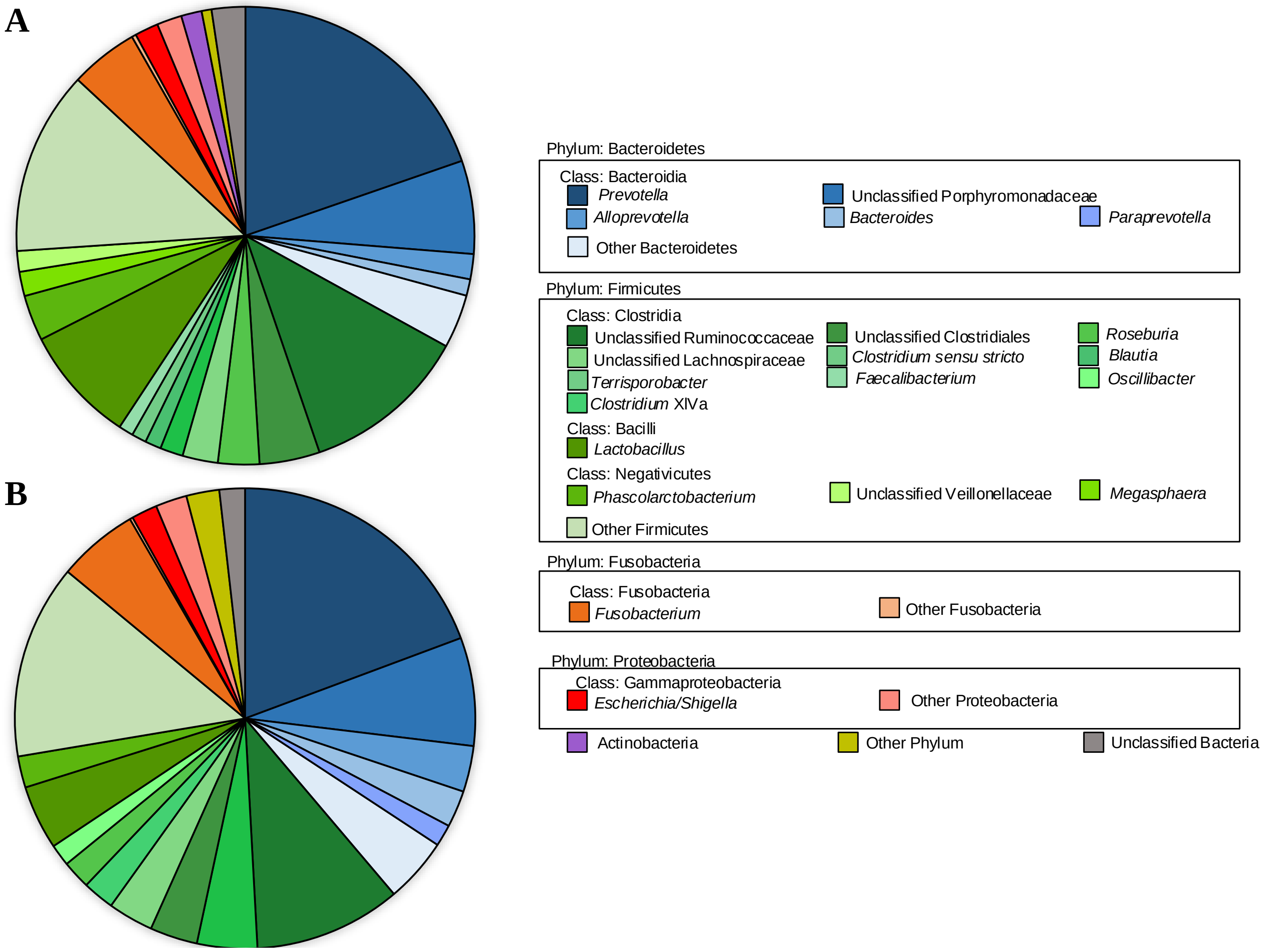

Supplementary Figure 1. Genus-level gut microbiota composition of the piglets. (**A**) The dominant genera in the 32 fecal samples of 8 piglets in T1. (**B**) The dominant genera in the 36 fecal samples of 9 piglets in T2.

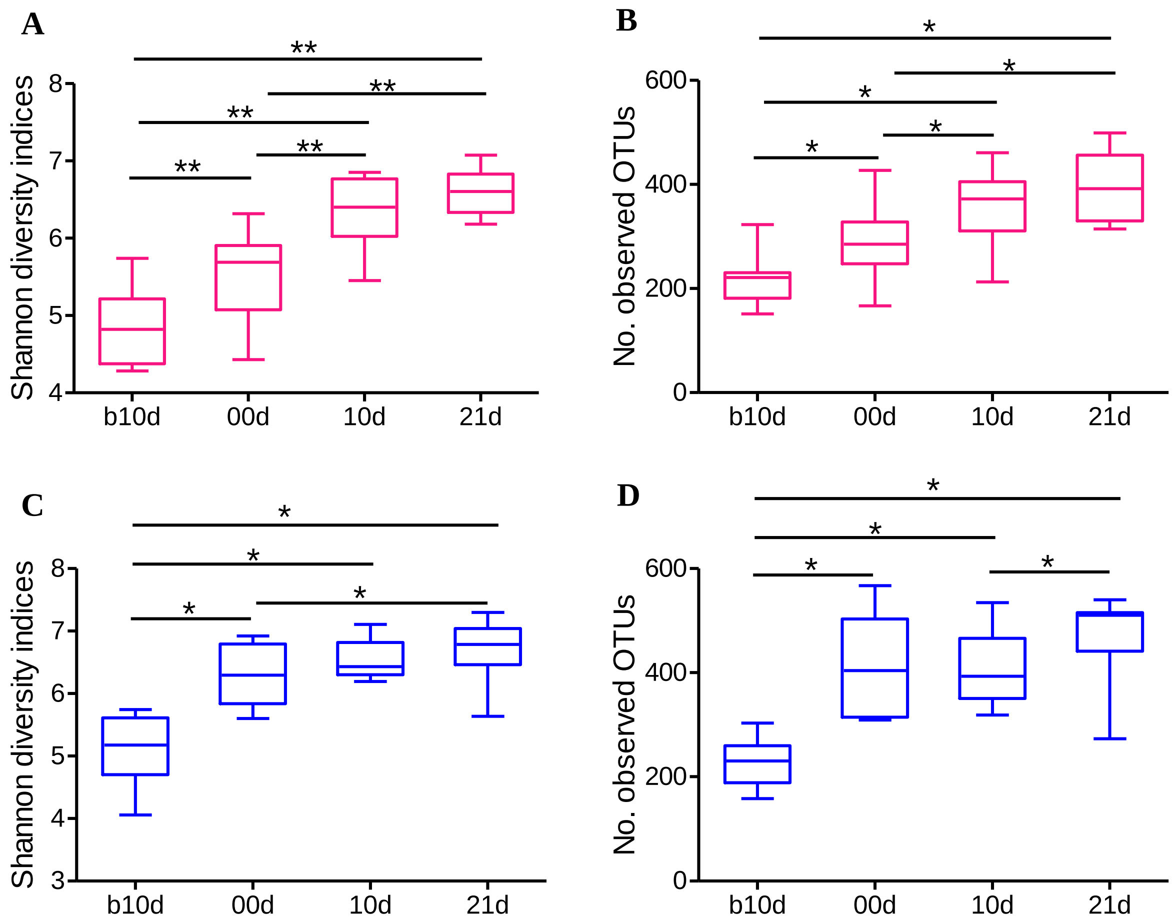

Supplementary Figure 2. Variations in alpha diversity of the piglets. (A) Comparisons of Shannon diversity indices between different age piglets in T1. (B) Comparisons of the number of observed OTUs between different age piglets in T1. (C) Comparisons of Shannon diversity indices between different age piglets in T2. (D) Comparisons of the number of observed OTUs between different age piglets in T2. All comparisons were tested by paired sample Wilcoxon signed-rank test and adjusted with FDR. Both alpha diversity metrics were calculated upon the rarified OTU subsets, using 13000 sequences per sample with 1,000 replications. In all panels, boxes represent the interquartile range (IQR) between the first and third quartiles. The lines inside boxes represent the median. Whiskers denote the lowest and highest values within 1.5 IQR from the first and third quartiles, respectively. Sample size: n=8 in T1, n=9 in T2. *, P<0.05; **, P<0.01; ***, P<0.001.

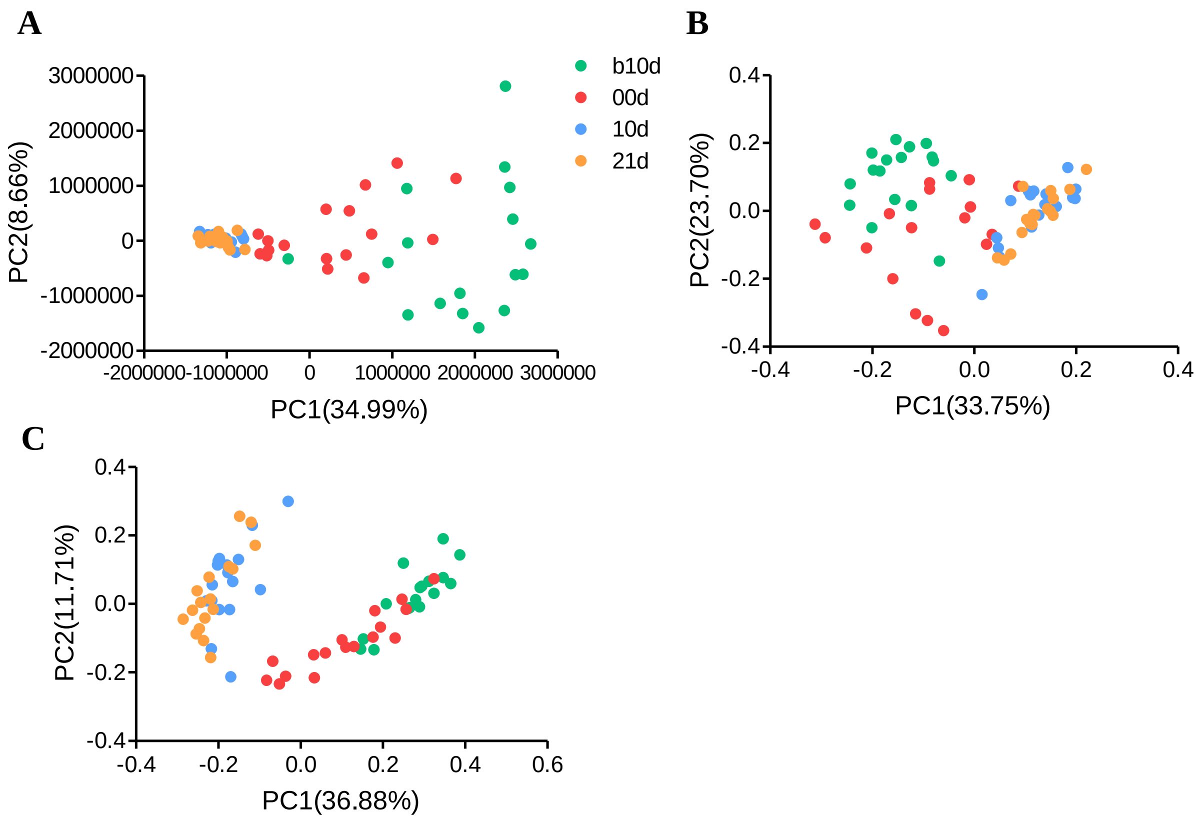

Supplementary Figure 3. Trajectory of the gut microbiota structure of each piglet across age based on other distances. (A) PCA. (B) PCoA plots based on weighted UniFrac distance. (C) PCoA plots based on unweighted UniFrac distance.

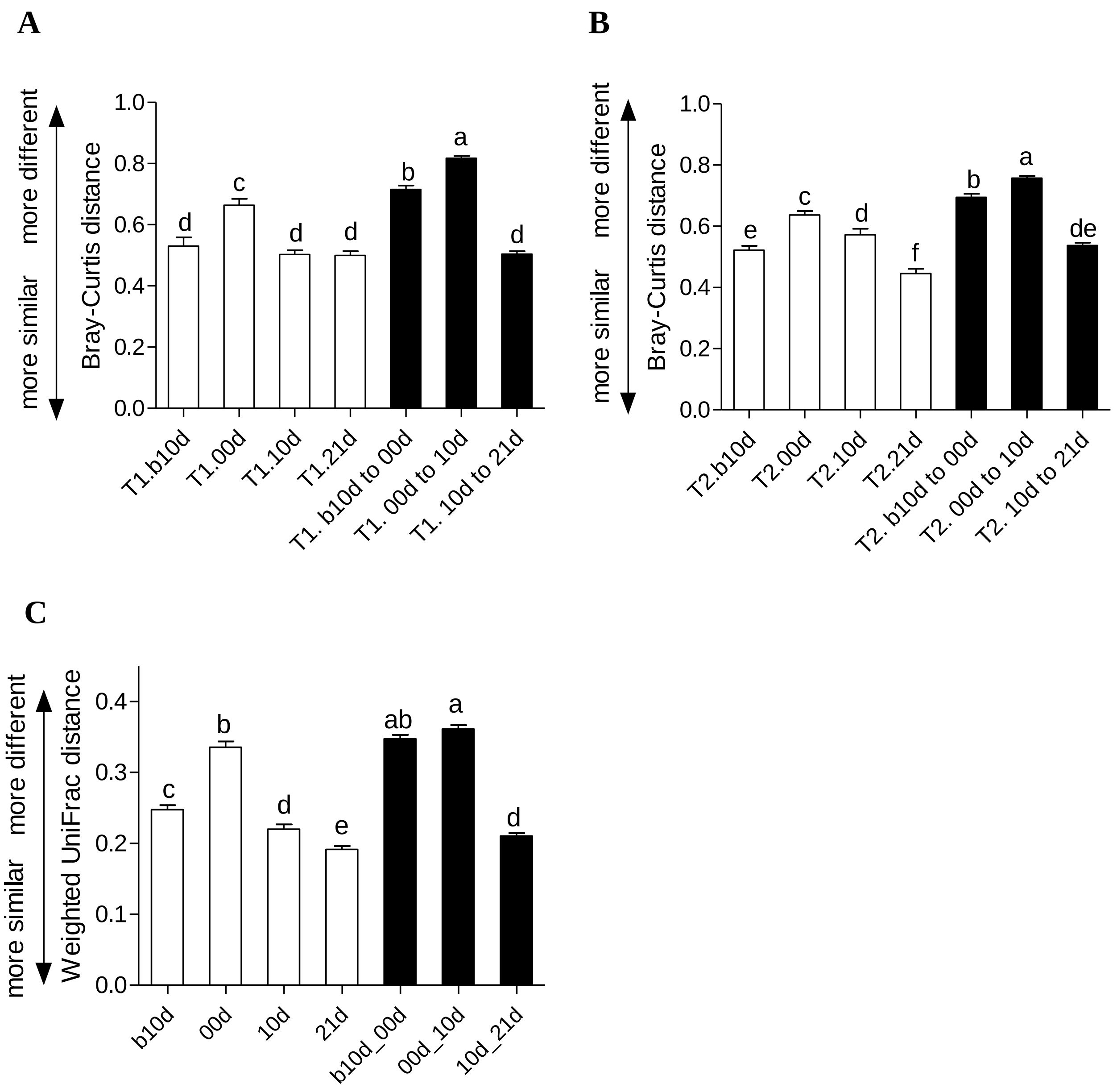

Supplementary Figure 4. Inter- and intraindividual variations of the gut microbiota of the piglets. (A) Inter- and intraindividual variations of the gut microbiota based on Bray-Curtis distance in T1. (B) Inter- and intraindividual variations of the gut microbiota based on Bray-Curtis distance in T2. (C) Inter- and intraindividual variations of the gut microbiota based on weighted UniFarc distance in all piglets. Mean values ±s.e.m are shown. Different letters above the bar denotes significant difference tested by Student's *t*-test with 1,000 Monte Carlo permutations.

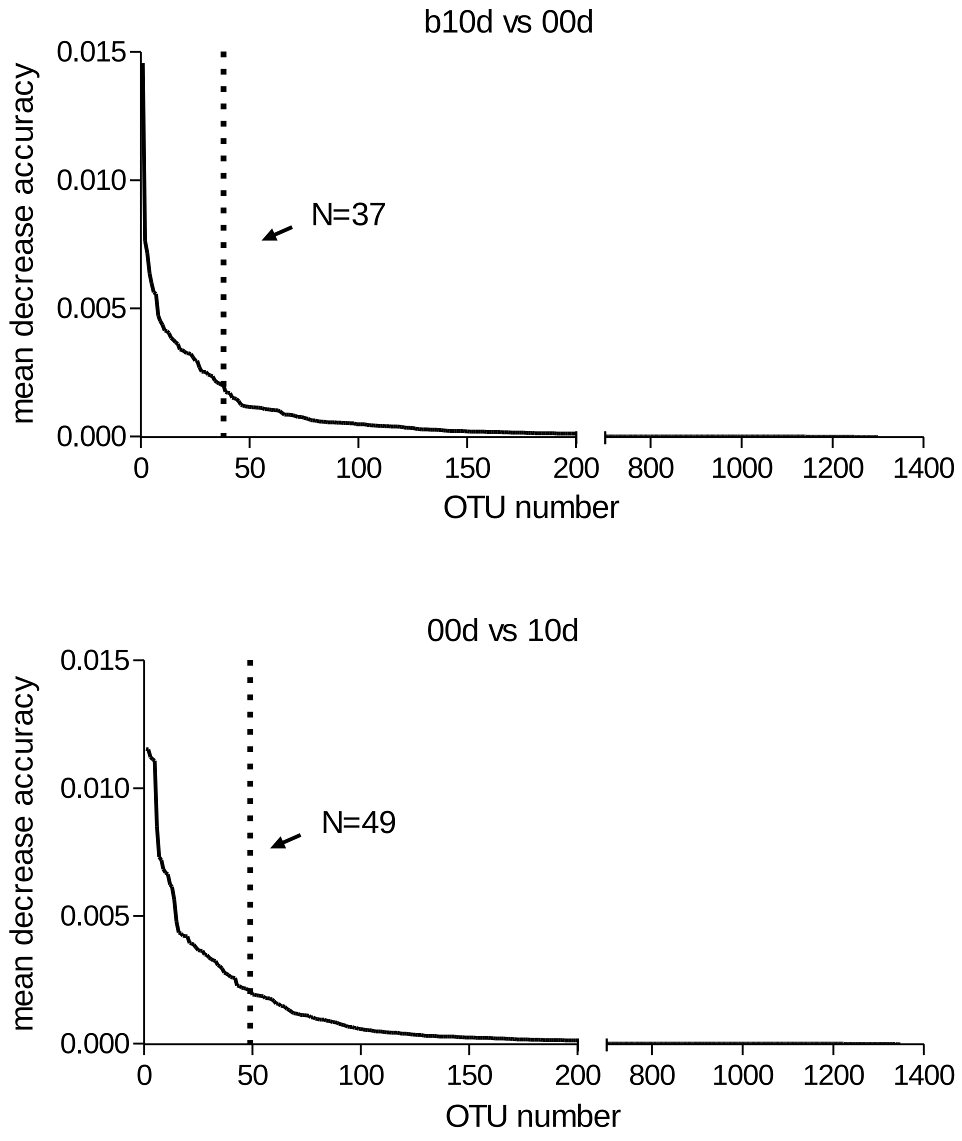

Supplementary Figure 5. 85 key OTUs were identified by applying Random Forest classification of their relative abundances in fecal samples, ranked in descending order of their feature accuracy of the models. N is the number of OTUs without which the accuracy of the model will decrease more than 0.2%. The 85 OTUs was the combination of N from 2 models.

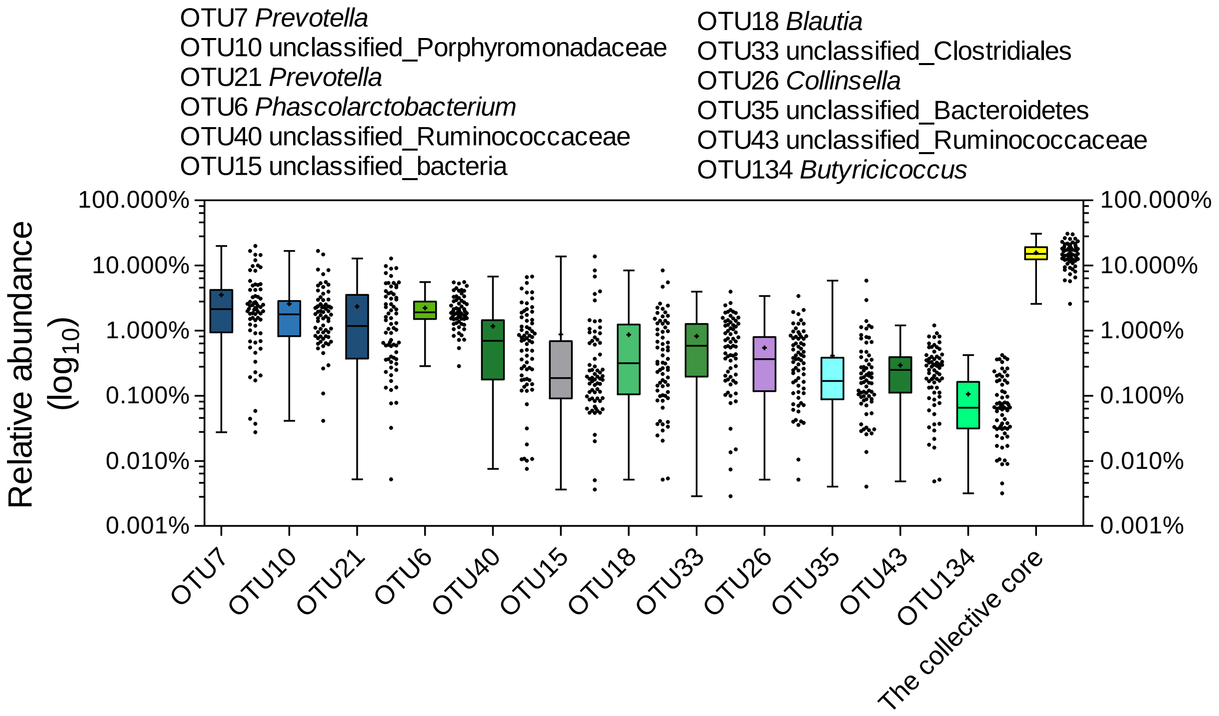

Supplementary Figure 6. The abundance distribution of the 12 OTUs and the collective core. Boxes represent the interquartile range (IQR) between the first and third quartiles. The lines and spots inside boxes represent the median and mean, respectively. Whiskers denote the lowest and highest values within 1.5 × IQR from the first and third quartiles, respectively.

1.2 Supplementary Tables

Supplementary Table 1. Ingredient and nutritional value analysis of basal diet.

Item	Ingredient （%）
Corn, yellow	56.50
Soybean meal	10.00
fermented soybean meal	6.00
extruded soybean	8.00
Fish meal	2.50
Dried whey	5.00
Good strong peptide	2.00
Glucose	2.50
Dicalcium phosphate	1.00
Limestone	0.50
salad oil	2.00
Premix feed[a]	4.00
Nutritional value analysis	
ME, Mcal/kg）	3.452
CP (%)	18.872
Lys (%)	1.381
Met (%)	0.566
Met+Cys(%)	0.838
Thr(%)	0.890
Trp(%)	0.253
Ca (%)	0.711
P (%)	0.612

a:Provided the following per kg of diet: rice bran power carrier 19.9g, Micro mineral package 3g, zinc oxide 2.5g, salt 3g, Lysine(98.5%) 4.5g, Methionine(99%) 2.7g, Threonine(98.5%) 1.5g, Tryptophan(98%) 0.4g, choline chloride(50%) 2g, Vitamin 0.5g.

Supplementary Table2. Impact of age on segregation of gut microbiota during suckling and weaning period.

Items	Bray Curtis (P^{a}-value)	Weighted UniFrac (P^{b}-value)
b10d *VS*. 00d	0.00012	0.00012
b10d *VS*. 10d	0.00012	0.00012
b10d *VS*. 21d	0.00012	0.00012
00d *VS*. 10d	0.00012	0.00012
00d *VS*. 21d	0.00012	0.00012
10d *VS*. 21d	0.0623	0.1646

a: PERMANOVA test based on Bray-Curtis distance of samples followed by FDR adjustment.
b: PERMANOVA test based on weighted UniFrac distance of samples followed by FDR adjustment.

Supplementary Table 3. Leave one out cross validation estimated error and baseline error of Random Forest models of each group paired.

Items	Estimated error	Baseline error	Ratio baseline error to estimated error
b10d *VS*. 00d	0.02941	0.5	17
00d *VS*. 10d	0	0.5	Inf

Supplementary Table 4. Taxonomy and dynamics information of 85 key OTUs.

OUT_ID	CAG group	Phylum	Family	Genus	b10d to 00d(P^a -value)	00d to 10d(P^b -value)	b10d to 10d(P^c -value)
OTU101	1	Firmicutes	Ruminococcaceae	*Butyricicoccus*	0.25	0.00127 * ↑	0.00088 * ↑
OTU83	1	Firmicutes	Ruminococcaceae	*Butyricicoccus*	0.375	0.00044 * ↑	0.00044 * ↑
OTU161	1	Firmicutes	Lachnospiraceae	*Lachnospiracea_incertae_sedis*	0.0625	0.00044 * ↑	0.00044 * ↑
OTU179	1	Bacteroidetes	Prevotellaceae	*Prevotella*	0.3125	0.00053 * ↑	0.00053 * ↑
OTU254	1	Firmicutes	Clostridiaceae 1	*Clostridium sensu stricto*	1	0.0009 * ↑	0.00088 * ↑
OTU145	1	Firmicutes	Ruminococcaceae	*Clostridium IV*	0.07813	0.00075 * ↑	0.00075 * ↑
OTU16	1	Firmicutes	Lactobacillaceae	*Lactobacillus*	0.00391 * ↑	0.00044 * ↑	0.00044 * ↑
OTU29	1	Firmicutes	Ruminococcaceae		0.01563 * ↑	0.00044 * ↑	0.00044 * ↑
OTU56	1	Bacteroidetes	Prevotellaceae	*Prevotella*	0.03125 * ↑	0.00044 * ↑	0.00044 * ↑
OTU61	1	Bacteroidetes	Prevotellaceae	*Prevotella*	0.00391 * ↑	0.00044 * ↑	0.00044 * ↑
OTU1206	1	Bacteroidetes	Prevotellaceae	*Prevotella*	1	0.00066 * ↑	0.00066 * ↑
OTU535	1	Bacteroidetes	Prevotellaceae	*Prevotella*	1	0.00009 * ↑	0.00009 * ↑
OTU1268	1	Bacteroidetes	Prevotellaceae	*Prevotella*	1	0.00009 * ↑	0.00009 * ↑
OTU651	1	Bacteroidetes	Prevotellaceae	*Prevotella*	0.3125	0.00063 * ↑	0.00063 * ↑
OTU173	1	Firmicutes			0.25	0.00066 * ↑	0.00066 * ↑
OTU844	1	Firmicutes			0.5	0.0021 * ↑	0.00131 * ↑
OTU177	1	Firmicutes	Ruminococcaceae		0.3125	0.00075 * ↑	0.00075 * ↑
OTU210	1	Actinobacteria	Coriobacteriaceae	*Olsenella*	0.3125	0.00096 * ↑	0.00096 * ↑
OTU1230	1	Firmicutes	Ruminococcaceae		0.01172 * ↑	0.00044 * ↑	0.00044 * ↑
OTU668	1	Firmicutes	Peptostreptococcaceae	*Clostridium XI*	0.06738	0.00066 * ↑	0.00066 * ↑
OTU215	1	Firmicutes	Lachnospiraceae	*Lachnospiracea_incertae_sedis*	0.00684 * ↑	0.00107 * ↑	0.00088 * ↑
OTU216	1	Firmicutes	Lachnospiraceae	*Lachnospiracea_incertae_sedis*	0.03906 * ↑	0.00044 * ↑	0.00044 * ↑
OTU122	1	Bacteroidetes	Prevotellaceae	*Prevotella*	0.96973	0.0009 * ↑	0.0009 * ↑
OTU24	1	Bacteroidetes	Prevotellaceae	*Prevotella*	0.16257	0.00075 * ↑	0.00075 * ↑
OTU54	1	Bacteroidetes	Prevotellaceae	*Prevotella*	0.33183	0.00044 * ↑	0.00044 * ↑
OTU19	1	Bacteroidetes	Prevotellaceae	*Prevotella*	0.19299	0.00107 * ↑	0.00088 * ↑
OTU104	2	Firmicutes	Lachnospiraceae	*Dorea*	0.00063 * ↑	0.83131	0.00063 * ↑
OTU98	2	Actinobacteria	Coriobacteriaceae		0.00063 * ↑	0.75831	0.00063 * ↑
OTU205	2	Firmicutes	Lachnospiraceae	*Clostridium XlVb*	0.00127 * ↑	0.09286	0.00127 * ↑
OTU456	2	Bacteroidetes	Bacteroidaceae	*Bacteroides*	0.00146 * ↑	0.63867	0.00146 * ↑
OTU268	2	Firmicutes	Lachnospiraceae		0.00406 * ↑	0.67912	0.00406 * ↑

OUT_ID	CAG group	Phylum	Family	Genus	b10d to 00d(P^a -value)	00d to 10d(P^b -value)	b10d to 10d(P^c -value)
OTU230	2	Bacteroidetes			0.00037 * ↑	0.12054	0.00586 * ↑
OTU256	2	Bacteroidetes	Porphyromonadaceae		0.00018 * ↑	0.04791 * ↓	0.01007 * ↑
OTU350	2	Bacteroidetes	Prevotellaceae	*Paraprevotella*	0.00073 * ↑	0.08325	0.03223 * ↑
OTU94	2	Bacteroidetes	Bacteroidaceae	*Bacteroides*	0.00131 * ↑	0.00241 * ↓	0.16882
OTU1053	3	Bacteroidetes	Prevotellaceae	*Prevotella*	0.0011 * ↑	0.01682 * ↑	0.0011 * ↑
OTU53	3	Firmicutes	Streptococcaceae	*Streptococcus*	0.0014 * ↑	0.0014 * ↑	0.00088 * ↑
OTU1191	3	Firmicutes	Ruminococcaceae		0.01007 * ↑	0.79459	0.01007 * ↑
OTU23	3	Firmicutes	Lachnospiraceae	*Clostridium XlVa*	0.00096 * ↑	0.05521	0.00096 * ↑
OTU44	3	Firmicutes	Lachnospiraceae	*Coprococcus*	0.0029 * ↑	0.01912 * ↑	0.0018 * ↑
OTU170	3	Actinobacteria	Coriobacteriaceae	*Senegalimassilia*	0.00073 * ↑	0.08402	0.00073 * ↑
OTU914	3	Firmicutes	Lachnospiraceae	*Blautia*	0.0014 * ↑	0.02772 * ↑	0.00088 * ↑
OTU62	3	Firmicutes	Lachnospiraceae	*Ruminococcus2*	0.02454 * ↑	0.00540 * ↑	0.00088 * ↑
OTU58	3	Firmicutes	Ruminococcaceae	*Faecalibacterium*	0.16257	0.00063 * ↑	0.00063 * ↑
OTU1244	3	Firmicutes	Acidaminococcaceae	*Phascolarctobacterium*	0.00127 * ↑	0.09286	0.00088 * ↑
OTU185	3	Firmicutes	Lachnospiraceae	*Clostridium XlVa*	0.00127 * ↑	0.83131	0.00105 * ↑
OTU136	3	Bacteroidetes			0.00481 * ↑	0.43476	0.00481 * ↑
OTU160	3	Firmicutes	Ruminococcaceae	*Butyricicoccus*	0.00073 * ↑	0.14766	0.00018 * ↑
OTU706	3	Firmicutes	Lachnospiraceae		0.00037 * ↑	0.13538	0.00037 * ↑
OTU246	3	Firmicutes	Erysipelotrichaceae		0.00073 * ↑	0.83131	0.00073 * ↑
OTU286	3	Firmicutes	Lachnospiraceae		0.00044 * ↑	0.00044 * ↑	0.00044 * ↑
OTU1	4	Firmicutes	Lactobacillaceae	*Lactobacillus*	0.09286	0.00044 * ↓	0.00044 * ↓
OTU214	4	Firmicutes	Veillonellaceae	*Mitsuokella*	0.00973 * ↑	0.00116 * ↓	0.00116 * ↓
OTU11	4	Firmicutes	Veillonellaceae	*Megasphaera*	0.03947 * ↓	0.00044 * ↓	0.00044 * ↓
OTU988	4	Firmicutes	Veillonellaceae	*Megasphaera*	0.08402	0.00044 * ↓	0.00044 * ↓
OTU48	4	Bacteroidetes	Prevotellaceae	*Prevotella*	0.00988 * ↓	0.00044 * ↓	0.00044 * ↓
OTU146	5	Proteobacteria	Sutterellaceae	*Sutterella*	0.00127 * ↓	0.01912 * ↓	0.00088 * ↓
OTU152	5	Bacteroidetes	Rikenellaceae	*Alistipes*	0.08402	0.00044 * ↓	0.00044 * ↓
OTU3	5	Proteobacteria	Enterobacteriaceae	*Escherichia/Shigella*	0.35596	0.00464 * ↓	0.0018 * ↓
OTU559	5	Synergistetes	Synergistaceae	*Cloacibacillus*	0.94339	0.00018 * ↓	0.00037 * ↓
OTU129	6	Bacteroidetes	Porphyromonadaceae	*Butyricimonas*	0.00226 * ↓	0.00018 * ↓	0.00044 * ↓
OTU28	6	Fusobacteria	Fusobacteriaceae	*Fusobacterium*	0.40743	0.00018 * ↓	0.00044 * ↓

OUT_ID	CAG group	Phylum	Family	Genus	b10d to 00d(P^a -value)	00d to 10d(P^b -value)	b10d to 10d(P^c -value)
OTU217	6	Bacteroidetes	Porphyromonadaceae	*Odoribacter*	0.10243	0.00096 * ↓	0.00096 * ↓
OTU140	6	Bacteroidetes	Porphyromonadaceae	*Parabacteroides*	0.0005 * ↓	0.00044 * ↓	0.00044 * ↓
OTU332	6	Bacteroidetes	Porphyromonadaceae	*Butyricimonas*	0.00044 * ↓	0.00098 * ↓	0.00044 * ↓
OTU13	7	Bacteroidetes	Porphyromonadaceae		0.04947 * ↑	0.00088 * ↓	0.00247 * ↓
OTU186	7	Firmicutes	Erysipelotrichaceae	*Sharpea*	0.72256	0.00044 * ↓	0.00037 * ↓
OTU369	7	Firmicutes			0.61915	0.00018 * ↓	0.00018 * ↓
OTU142	7	Bacteroidetes	Porphyromonadaceae		0.87672	0.00009 * ↓	0.00009 * ↓
OTU174	7	Bacteroidetes	Porphyromonadaceae		0.17729	0.00018 * ↓	0.00066 * ↓
OTU22	7	Bacteroidetes	Porphyromonadaceae		0.61915	0.00066 * ↓	0.00066 * ↓
OTU2	7	Fusobacteria	Fusobacteriaceae	*Fusobacterium*	0.00085 * ↓	0.00044 * ↓	0.00044 * ↓
OTU42	7	Bacteroidetes	Porphyromonadaceae		0.24614	0.00066 * ↓	0.00066 * ↓
OTU17	8	Firmicutes	Ruminococcaceae		0.14879	0.00126 * ↓	0.01122 * ↓
OTU75	8	Bacteroidetes	Porphyromonadaceae		0.56949	0.00066 * ↓	0.00018 * ↓
OTU112	9	Firmicutes	Peptostreptococcaceae	*Peptostreptococcus*	0.00018 * ↓	0.25000	0.00018 * ↓
OTU424	9	Proteobacteria	Sutterellaceae	*Sutterella*	0.0008 * ↓	0.03906 * ↓	0.0008 * ↓
OTU139	9	Bacteroidetes	Porphyromonadaceae	*Falsiporphyromonas*	0.0008 * ↓	0.0625	0.0008 * ↓
OTU79	9	Bacteroidetes	Bacteroidaceae	*Bacteroides*	0.0034 * ↓	0.04947 * ↓	0.00088 * ↓
OTU8	9	Bacteroidetes	Prevotellaceae	*Prevotella*	0.0021 * ↓	0.30879	0.0021 * ↓
OTU110	10	Firmicutes	Lachnospiraceae		0.00214 * ↑	0.03516 * ↓	0.01075 * ↑
OTU187	10	Firmicutes			0.00037 * ↑	0.00641 * ↓	0.12305
OTU276	10	Bacteroidetes	Prevotellaceae		0.00037 * ↑	0.11774	0.49219
OTU221	10	Firmicutes	Erysipelotrichaceae	*Turicibacter*	0.00018 * ↑	0.00018 * ↓	0.81250
OTU258	10	Proteobacteria	Campylobacteraceae	*Campylobacter*	0.00406 * ↑	0.00159 * ↓	0.04187 * ↓

a: OTUs that were significantly changed between b10d and 00d. ↑ significantly increased than b10d, ↓ significantly decreased.
b: OTUs that were significantly changed between 00d and 10d. ↑ significantly increased than 00d, ↓ significantly decreased.
c: OTUs that were significantly changed between b10d and 10d. ↑ significantly increased than b10d, ↓ significantly decreased.

Supplementary Table 5. The 12 most prevalent OTUs identified in this study

OUT ID	Phylum	Genus	Proportion in total sequences (%)
OTU7	Bacteroidetes	Prevotella	3.545%
OTU21	Bacteroidetes	Prevotella	2.347%
OTU10	Bacteroidetes	unclassified_Porphyromonadaceae	2.571%
OTU35	Bacteroidetes	unclassified_Bacteroidetes	0.412%
OTU6	Firmicutes	Phascolarctobacterium	2.231%
OTU40	Firmicutes	unclassified_Ruminococcaceae	1.163%
OTU43	Firmicutes	unclassified_Ruminococcaceae	0.294%
OTU33	Firmicutes	unclassified_Clostridiales	0.826%
OTU15	Firmicutes	unclassified_bacteria	0.879%
OTU134	Firmicutes	Butyricicoccus	0.106%
OTU18	Firmicutes	Blautia	0.870%
OTU26	Actinobacteria	Collinsella	0.546%
Core OTUs			15.79%
